# Mesoporous Polydopamine Loaded Pirfenidone Target to Fibroblast Activation Protein for Pulmonary Fibrosis Therapy

**DOI:** 10.3389/fbioe.2022.920766

**Published:** 2022-07-22

**Authors:** Qi Fang, Shaoyu Liu, Jiangyu Cui, Ruiyue Zhao, Qian Han, Peng Hou, Youcai Li, Jie Lv, Xiaoyao Zhang, Qun Luo, Xinlu Wang

**Affiliations:** ^1^ Department of Nuclear Medicine, The First Affiliated Hospital of Guangzhou Medical University, Guangzhou, China; ^2^ State Key Laboratory of Respiratory Diseases, Guangzhou Institute of Respiratory Diseases, The First Affiliated Hospital of Guangzhou Medical University, Guangzhou, China

**Keywords:** mesoporous polydopamine, fibroblast activation protein, fibroblast activation protein inhibitor, pulmonary fibrosis, antifibrosis therapy

## Abstract

Recently, fibroblast activation protein (FAP), an overexpressed transmembrane protein of activated fibroblast in pulmonary fibrosis, has been considered as the new target for diagnosing and treating pulmonary fibrosis. In this work, mesoporous polydopamine (MPDA), which is facile prepared and easily modified, is developed as a carrier to load antifibrosis drug pirfenidone (PFD) and linking FAP inhibitor (FAPI) to realize lesion-targeted drug delivery for pulmonary fibrosis therapy. We have found that PFD@MPDA-FAPI is well biocompatible and with good properties of antifibrosis, when ICG labels MPDA-FAPI, the accumulation of the nanodrug at the fibrosis lung *in vivo* can be observed by NIR imaging, and the antifibrosis properties of PFD@MPDA-FAPI *in vivo* were also better than those of pure PFD and PFD@MPDA; therefore, the easily produced and biocompatible nanodrug PFD@MPDA-FAPI developed in this study is promising for further clinical translations in pulmonary fibrosis antifibrosis therapy.

## 1 Introduction

Pulmonary fibrosis is the terminal of many interstitial lung diseases (ILDs), it damages the normal lung structure, causes lung function decay, impairs the quality of life, and even leads to death by fibroblasts activated aberrantly and extracellular matrix (ECM) accumulated excessively (Rockey et al., 2015; Deng et al., 2020; Lai et al., 2021). Idiopathic pulmonary fibrosis (IPF) is a very common ILD, manifesting progressive pulmonary fibrosis with unclear etiology, the middle survival time of IPF is 2–5 years (Fernández Pérez et al., 2010; Ley et al., 2011; Nathan et al., 2011; Raghu et al., 2011). Nowadays, the critical treatment for IPF is antifibrosis therapy, for example, pirfenidone (PFD). PFD had finished the phase three trial, represented a good effect on patients with IPF, but often comes along with gastrointestinal (GI) adverse events (AE), which may be brought by the oral administration (King et al., 2014; Lancaster et al., 2017).

Activated fibroblast is a key cell in pulmonary fibrosis pathogenesis, and fibroblast activation protein (FAP), a 97 kDa type II transmembrane protein, with dipeptidyl peptidase activity as one kind of serine protease, only overexpressed on the membrane of aberrantly activated fibroblast, had been proved that could be found at the remolding area of IPF and cancer (Garin-Chesa et al., 1990; Acharya et al., 2006; Keane et al., 2013). So FAP has become the target of pulmonary fibrosis diagnosis and therapy, which interests many researchers (Wenlong et al., 2015; Lin et al., 2019; Wu et al., 2020; Bergmann et al., 2021; Rohrich et al., 2022). It had been reported that FAP inhibitor (FAPI) labeled by radionuclide could reveal the FAP expression in patients with ILD, (Bergmann et al., 2021; Rohrich et al., 2022) positron emission tomography computed tomography (PET/CT) used FAPI as a tracer maybe is a promising imaging modality for ILDs to detect lesion earlier than CT, because CT only focuses on the change of structure, but FAPI PET/CT can observe the metabolism of the fibroblast before the change of anatomy. At the same time, Ma et al. have proved that bioluminescent probe target to FAP successfully indicated that the expression of FAP in the lung of the pulmonary fibrosis mice was increased (Lin et al., 2019). FAP is also has been proved that can play a role in antifibrosis therapy, Egger et al. (2017) reported that FAP inhibitor PT100 manifests antifibrosis properties in pulmonary fibrosis mice induced by bleomycin (BLM). Getting et al. found that FAP stimulates the activation of fibroblast (Wu et al., 2020). But in contrast, Kimura et al. found that the lack of FAP accelerates fibrosis. Meanwhile, Fan et al. (2016) found that FAP can accelerate the degradation and clearance of collagen. Therefore, the real role that FAP plays in fibrosis remains a mystery, but we may base the specific membrane protein to develop a class of target drug delivery nano.

There has been no nanodrug target to FAP for antifibrosis therapy before. Recently, nanodrugs that utilize nanoparticles as drug delivery carriers for targeting lesions, releasing drugs, improving pharmacokinetics, biocompatibility, and bioavailability interested people greatly (George et al., 2019; Altinoglu and Adali, 2020; Maghsoudi et al., 2020; Liu et al., 2021; Abd Al-Jabbar et al., 2022; Sultan et al., 2022). The mesoporous polydopamine (MPDA) is one of the most popular nanoparticles because of its intrinsic biocompatibility, facile preparation, and various easily modified surface groups (Seth et al., 2020; Lin et al., 2021; Zhu et al., 2021). Therefore, the nanodrug based on MPDA targets to FAP administrated by intravenous (IV) is one of the promising approaches for antifibrosis therapy without GI AE.

**SCHEME 1:**
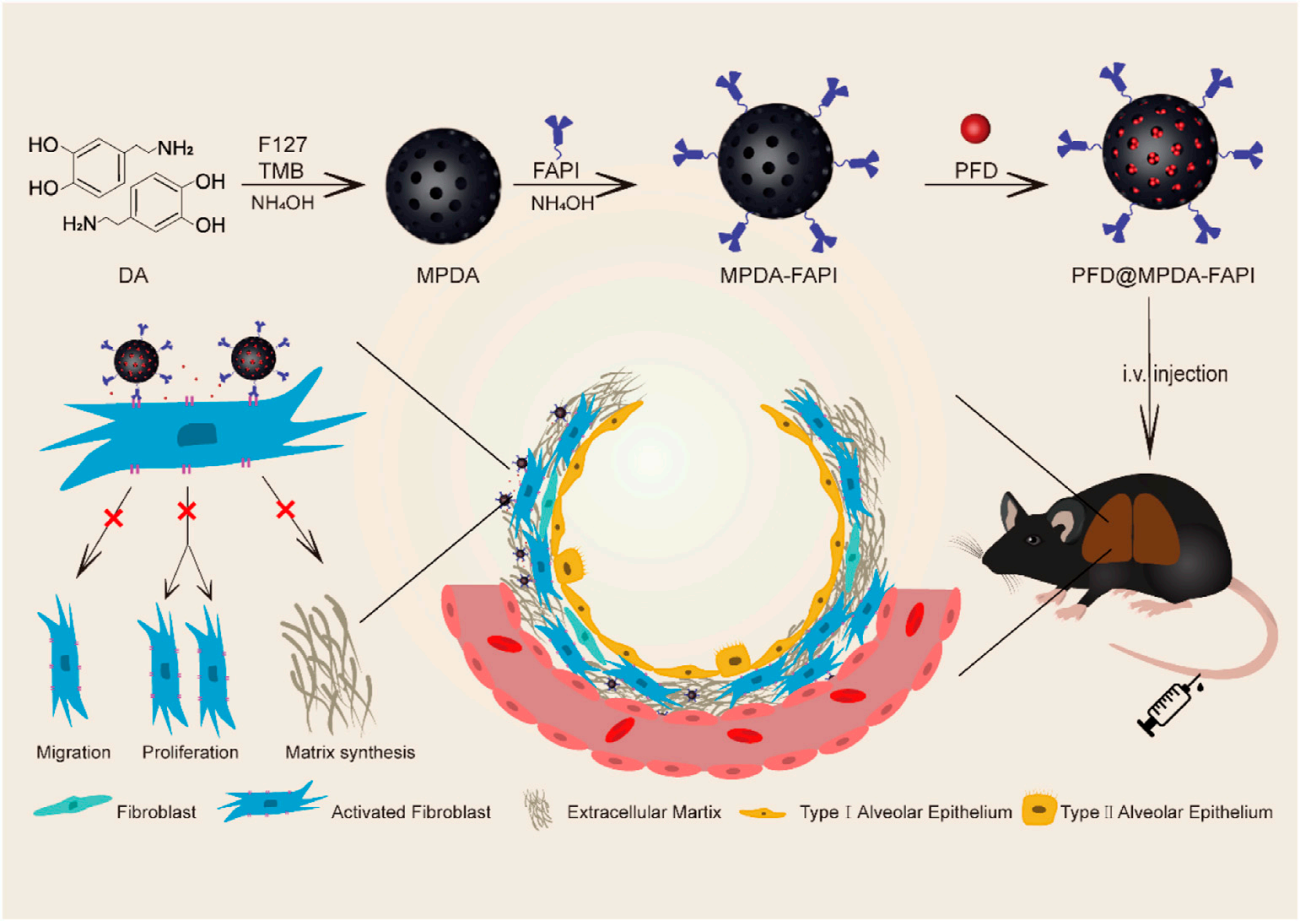
Schematic illustration of the synthetic procedures and therapy mechanisms of PFD@MPDA-FAPI.

Here, we develop a nanodrug, PFD@MPDA-FAPI, based on MPDA, loading with PFD, and linking FAPI for antifibrosis therapy by FAP targeting drug delivery (Scheme 1), it is well biocompatibility and performs well at antifibrosis. MPDA was synthesized from dopamine hydrochloride with F127 and TMB as templates and then to remove template; NH_2_-PEG_2_-FAPI was conjugated to MPDA *via* condensation. PFD was loaded in the mesoporous on MPDA-FAPI. Compared to pure PFD and PFD@MPDA, PFD@MPDA-FAPI could target activated fibroblast *in vitro* and fibrosis lung *in vivo*, as well as improving the antifibrosis efficacy. This promising strategy demonstrates a new avenue for antifibrosis therapy.

## 2 Experimental Section

### 2.1 Materials

Dopamine hydrochloride (Aladdin, Shanghai, China) was used to synthesize MPDA; 1,3,5-trimethyl benzene (TMB) and F127 (Macklin, Shanghai, China) were used as the template. NH_2_-PEG-FAPI (Tanzhenbio, Nanchang, China) was used for FAP targeting. Pirfenidone (Aladdin, Shanghai, China) is an antifibrosis drug. In immunohistochemical (IHC) staining, FN antibody (ab2413, Invitrogen) and FAP antibody (PA5-99458, Invitrogen) diluted 1:200, and α-SMA antibody (19245S, CST) diluted 1:800. For Western blot, FN antibody and FAP antibody diluted 1:1,000 was used. All other reagents were obtained from Aladdin.

### 2.2 Synthesis of MPDA and MPDA-FAPI

MPDA was synthesized by a facile approach. In short, 150 mg of dopamine hydrochloride was dissolved in 3 ml of deionized water, and 100 mg of F12 was dissolved in 3 ml of ethanol. The F127 solution was added to the dopamine solution, then added 160 μl of TMB, followed by 2 min of ultrasonic mixing, then 375 μl of 28% NH_4_OH was added to the solution finally. After magnetic stirring for 2 h of the mixed solution, MPDA was obtained by centrifugal washing three times and resuspended in 1 ml deionized water. After drying, 1 ml MPDA solution weighs 1.5–2 mg. MPDA-FAPI was synthesized as follows. Then, 28% NH_4_OH was used to adjust the pH of 3 ml MPDA solution to 9, then added 2 mg FAPI-NH_2_-PEG, then the MPDA-FAPI was obtained by centrifugal washing after magnetic stirring at room temperature overnight.

### 2.3 Drug Loading and Release

First, 10 mg PFD was dissolved in 2 ml of methanol, then added PFD solution into 500 μl MPDA solution, magnetic stirring overnight and then centrifugally washing, finally resuspended the sediment in 1 ml deionized water to obtain MPDA@PFD. We tested the ultraviolet-visible (UV-Vis) absorbance of different concentrations of PFD to draw a standard curve of PFD, which is used for calculating the PFD concentration of nanodrug. The loading capacity is the weight ratio of PFD in MPDA@PFD. The 500 μl MPDA@PFD was sealed in a dialysis bag and put in a 15 ml tube with 2 ml PBS (pH 7.4) buffer solution, the mixture was shaken for 72 h a speed of 150 rpm. Periodically remove 0.5 ml of dialysate with a pipette gun and immediately add 0.5 ml of fresh PBS buffer to keep the constant total volume of the solution. After 72 h, the drug release was measured by the UV-Vis spectrophotometer.

### 2.4 Characterization

The morphology of MPDA and MPDA-FAPI were observed by using a transmission electron microscope (TEM). Firstly, prepared MPDA and MPDA-FAPI were dripped on the copper net, then dried and placed under the transmission electron microscope. The hydrodynamic diameter and the distribution of MPDA and MPDA-FAPI used dynamic light scattering (DLS) and zeta potential were measured by the laser nanometer particle size analyzer. The Fourier transform infrared (FTIR) spectrum and UV-vis spectrum of samples were obtained as follows. The proper amount of samples (DA, MPDA, and MPDA-FAPI) were grinded and pressed with KBr, respectively, then the prepared samples were detected by FTIR spectrometer, the scanning wavelength range was 500–4000 cm^−1^. The appropriate number of samples (PFD, MPDA, and MPDA@PFD) were uniformly dispersed in the deionized water, using the UV-Vis spectrophotometer to record the spectrum of samples within the range of 200–600 nm.

### 2.5 *In Vitro* Experience

#### 2.5.1 Cell Culture

HFL1 (human fetal lung fibroblast, Procell CL-0106) was provided by Procell Life Science and Technology Co., cultured in Ham’s F-12K with 10% fetal bovine serum (FBS) and 1% penicillin-streptomycin, cells were kept in a humidified atmosphere containing 5% CO_2_ at 37°C.

#### 2.5.2 Cytotoxicity and Blood Compatibility

For the cytotoxicity assay *in vitro*, HFL1 was first seeded into 96-well plates at a density of 5,000 cells per well overnight. Then, the original medium is then taken away and replaced with a fresh one containing various concentrations of nanoparticles and incubated for 24 h. The concentration of MPDA and MPDA-FAPI were 10–500 μg/ml. Finally, 100 μl, 0.1 μl/ml CCk8 solution was added to determine the relative cell viabilities among different concentration of nanoparticles treated groups as well as the untreated control.

Then, 200 μl 16% red blood cell suspension was incubated with MPDA and MPDA-FAPI (0.01 mg/ml), respectively, then centrifuged for 5 min, 1,000 × g at pre-set time points (1, 3, 6, 9, 18, and 24 h) to obtain supernatant. The Microplate Reader measured the absorbance of each supernatant at 540 nm to calculate the hemolysis rate. Whole blood was centrifuged with 1,000 × g for 5 min, then the lower red blood cells were collected, and incubated with MPDA-FAPI (0.01 mg/ml) for 15 min, then centrifuged to collect the red blood cell, then washed with PBS, and added 4% paraformaldehyde for 1 h. The fixed red blood cells were dehydrated with 70, 85, 95, and 100% ethanol, respectively, for 10 min. After the samples were dried, the samples were sprayed with gold, and the cell morphology was observed by scanning electron microscope (SEM).

#### 2.5.3 Cellular Uptake Analyzed

FITC-MPDA and FITC MPDA-FAPI were prepared for cellular uptake experiment, obtained by stirring MPDA or MPDA-FAPI (1 ml) with FITC (300 μg) overnight, followed with centrifugally washed, and resuspended in 1 ml deionized water. Cellular uptake of FITC-MPDA and FITC-MPDA-FAPI were analyzed by endocytosis experiments. Specifically, HFL1 cells were seeded into a 24-well plate with a density of 5 × 10^4 cells/holes, cultured overnight, and half of the holes were treated with 10 ng/ml TGF-β for 48 h. Then added to a complete medium containing 50 μg/ml FITC-MPDA or FITC-MPDA-FAPI. After incubating for 0.5, 2, and 4 h, the cells in each group were washed with PBS to remove the free material, digested with trypsin, and centrifuged to collect the cells. Finally, cells were resuspended by 200 μl PBS and detected by flow cytometry, the fluorescence intensity was measured by Flow JO 7.6.1 software.

The endocytosis of MPDA-FITC or MPDA-FAPI-FITC was also observed by confocal laser scanning. Specifically, HFL1 cells were first seeded in a cell culture dish with a density of 2 × 10^5 cells per dish and then cultured overnight; then, two dishes of cells were treated with TGF-β for 48 h and finally added to a complete medium containing 50 μg/ml FITC-MPDA or FITC-MPDA-FAPI. The cells were then washed with PBS after incubation for 4 h, then the cells were immobilized with 4% paraformaldehyde for 30 min and washed with PBS three times, the cell nucleus was stained with 1 ml DAPI in each dish for 15 min, and the cells were washed with PBS three times, and 2 ml PBS was added to each dish to keep the cell morphology, finally cells were observed and photographed with a laser confocal microscope.

#### 2.5.5 Cell Proliferation Inhabitation

MTT assay was used to investigate the effects of MPDA@PFD and PFD@MPDA-FAPI on HFL1 with and without TGF-β treated. HFL1 cells were seeded into a 96-well plate at a density of 5,000 cells per hole and then cultured overnight. The original medium is then taken away and replaced with fresh complete media containing different concentrations of nanomaterials, the concentration of PFD in MPDA@PFD and MPDA-FAPII@PFD was in the range of 10–100 μg/ml, 50 μl, 1 mg/ml. MTT solution was then added to determine the relative cell viabilities among different concentration of nanoparticles treated groups as well as the untreated control.

#### 2.5.6 Cell Scratches Experiment

HFL1 cells were seeded into a 24-well plate with a density of 5 × 10^^4^ cells per hole and cultured overnight, then treated with 10 ng/mg TGF-β for 48 h and scratched the bottom of the well to create artificial gaps in the cell. Then the cells were divided into three groups, incubated with 50 μg/ml MPDA@PFD, PFD@MPDA-FAPI, and PBS especially for 12 h, then washed with PBS, cultured in DMEM (serum-free), and photographed the gaps at the same position every different time (0, 6, and 24 h) by an inverted microscope, ImageJ software was used to measure and analyze the scratch area.

### 2.6 *In Vivo* Experience

#### 2.6.1 Pulmonary Fibrosis Animal Model

C57BL/6 male mice (6–8 weeks, w ≈ 25 g) were used for the study. Animals were kept in a good environment. Drinking water and food were freely available. Mice were lightly anesthetized with 1.5% pentobarbital sodium, and BLM (2 mg/kg, YuanYe, Shanghai) was administered transtracheal *via* a laryngoscope (Yuyan, Shanghai). Micro CT (ALOKA LCT-200) was performed after 6 days to confirm the interstitial lung disease (Supplementary Figure S5).

#### 2.6.2 *In Vivo* Imaging

MPDA and MPDA-FAPI are prepared for ICG/NIR imaging. The nanodrug labeled by ICG was obtained by stirring MPDA or MPDA-FAPI (1 ml) and 1 ml 1 mg/ml ICG overnight, followed by centrifugally washed and resuspended in 1 ml deionized water. ICG-MPDA and ICG-MPDA-FAPI were administrated *via* tail vein, and *in vivo* imaging was obtained after 10 min, 1, 3, 5, and 8 h administration of the pulmonary fibrosis mice. After 8 h imaging, the heart, liver, spleen, lungs, and kidneys were resected to quantify the radiant efficiency *in vitro*.

#### 2.6.3 *In Vivo* Antifibrosis Therapy

Three healthy mice as the control group. Then, 15 pulmonary fibrosis mice were divided into five groups randomly, named NaCl, MPDA, PFD, PFD@MPDA, and PFD@MPDA-FAPI groups especially. Except for the control group, other groups of mice were pulmonary fibrosis, after 7 days induced by BLM, each group of mice was IV administrated with the corresponding drugs every 2 days regularly. In NaCl and MPDA groups, mice were IV injected with 120 μl NS and 120 μl MPDA solution, respectively. In the PFD, PFD@MPDA, and PFD@MPDA-FAPI groups, the dosage of PFD was 6 mg/kg. After three times of therapy, all the mice were sacrificed, right lungs were collected for WB analyses, and left lungs were collected for histological analyses.

#### 2.6.4 Alveolitis and Fibrosis Grading

In histological analyses, HE staining and Masson staining were performed for grading alveolitis and fibrosis, the grading is according to the method of Szapiel et al. (1979). Alveolitis was evaluated with the HE stained sections and was graded using the criteria as follows: none (0), no alveolitis; mild (1+), thickening of the alveolar septum by a mononuclear cell infiltrate, with involvement limited to focal, pleural-based lesions occupying less than 20% of the lung and with good preservation of the alveolar architecture; moderate (2+), more widespread alveolitis involving 20–50% of the lung, although still predominantly pleural based; severe (3+), diffuse alveolitis involving more than 50% of the lung, with occasional consolidation of air spaces by the intra-alveolar mononuclear cells and some hemorrhagic areas within the interstitium and/or alveolus. The extent of fibrosis in these sections was graded using the following criteria: none (0), no evidence of fibrosis; mild (1+), focal regions of fibrosis involving less than 20% of the lung. Fibrosis involved the pleura and the interstitium of the subpleural parenchyma with some distortion of alveolar architecture; moderate (2+), more extensive fibrosis involving 20–50% of the lung and fibrotic regions mostly extending inward from the pleura and still focal; severe (3+), widespread fibrosis, involving more than 50% of the lung. Confluent lesions with extensive derangement of parenchymal architecture, including cystic air spaces lined by cuboidal epithelium.

#### 2.6.5 IHC Staining

FN antibody and FAP antibody were diluted to 1:200, and α-SMA antibody was diluted to 1:800 for IHC staining. Image-Pro Plus was used to calculate the average optical density (AOD) of FN, FAP, and α-SMA among different groups.

#### 2.6.6 Western Blot Analyses

The expression of FN and FAP in different groups was analyzed by WB. FN antibody and FAP antibody are diluted to 1:1,000 for WB.

## 3 Result and Discussion

### 3.1 Synthesis and Characterization of MPDA, PFD@MPDA, and PFD@MPDA-FAPI

The synthetic procedures of MPDA, MPDA-FAPI, and PFD@MPDA-FAPI nanoparticles are depicted in Scheme 1. First, the soft templating method was used to synthesize MPDA. All these nanoparticles were obtained in the aqueous solution. As for the production of MPDA, F127, and TBM were chosen as templates, ethanol, and ammonia solution were used for cosolvent and the catalyst in the polymerization of DA, then *via* the π–π stacking interactions, PDA was self-assembled on the templates, MPDA NPs were finally obtained by removing of templates (Wu et al., 2019; Lin et al., 2021). Second, NH_2_-PEG_2_-FAPI was conjugated to MPDA *via* condensation of the amine groups on NH_2_-PEG_2_-FAPI and the dihydroxy indole groups on MPDA to form MPDA-FAPI. Finally, PFD was loaded onto MPDA and MPDA-FAPI by magnetic stirring to form PFD@MPDA and PFD@MPDA-FAPI.

**FIGURE 1:**
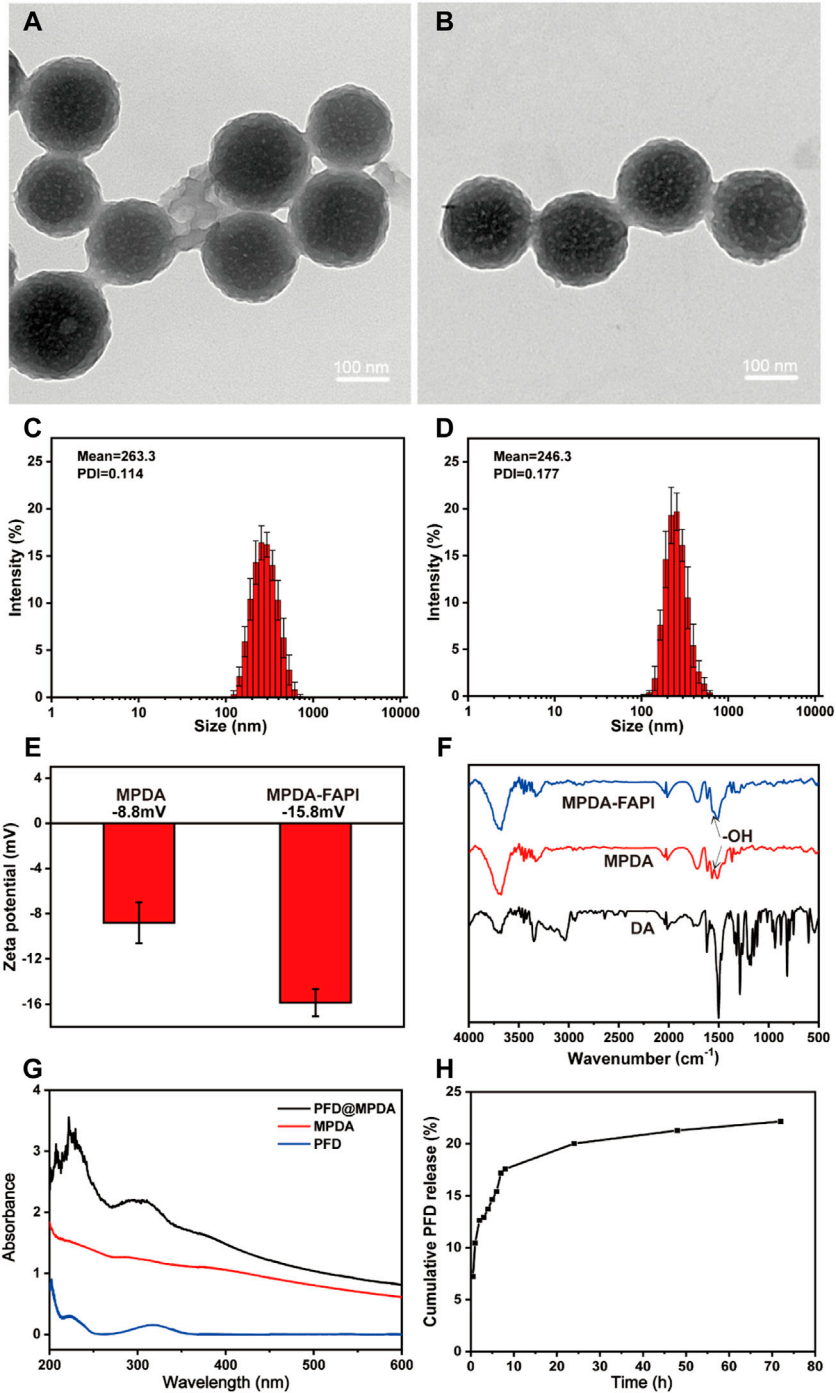
TEM images of **(A)** MPDA and **(B)** MPDA-FAPI. Hydrodynamic diameters of **(C)** MPDA and **(D)** MPDA-FAPI. **(E)** Zeta potential of MPDA and MPDA-FAPI. **(F)** FTIR absorption spectrum of DA, MPDA, and MPDA-FAPI. **(G)** UV/Vis absorption spectrum of MPDA, PFD, and PFD@MPDA. **(H)** PFD released from PFD@MPDA at pH 7.4 for 72 h.

TEM observations confirmed that MPDA and MPDA-FAPI NPs manifest the well-defined spherical morphology and mesoporous structure, as well as uniform size distribution (Figures 1A,B), and the huge surface area created by mesoporous structure, is suitable for drug loading. The average diameters of MPDA and MPDA-FAPI NPs are all about 200 nm. DLS measurement also demonstrates that no aggregation occurs of these nanoparticles, the average hydrodynamic diameters of MPDA and MPDA-FAPI nanoparticles are ≈263 and 246 nm, respectively (Figures 1C,D). Compared to the diameter of the dried sample used in TEM measurement, the hydrodynamic diameter is larger. The tiny variety of the nanoparticles’ diameter between TEM and DLS results may occur by swelling effect, which is common in polymer material. The nanoscale diameter is safe for IV administration without pulmonary embolism risk. Meanwhile, the zeta potentials of MPDA and MPDA-FAPI nanoparticles are −8.8 and −15.8 mV, respectively (Figure 1E), the lower charge of MPDA-FAPI confirmed the conjugation of MPDA and the negatively charged NH_2_-PEG_2_-FAPI. FTNR spectrum can also prove the conjunction of MPDA and FAPI, the condensation of the amine groups on NH_2_-PEG_2_-FAPI with the hydroxy indole groups on MPDA, consuming the hydroxyl, because of hydroxyl on MPDA manifests a characteristic absorption band at 1,500 cm^−1^, hence, the lower absorbance of MPDA-FAPI at 1,500 cm^−1^ proved the condensation of MPDA and NH_2_-PEG_2_-FAPI (Figure 1F).

### 3.2 Drug Loading and Release Properties

The UV-Vis absorption spectrum of PFD@MPDA clearly shows the characteristic absorption band of PFD at 319 nm (Figure 1G). Based on the standard curve of PFD (Supplementary Figure S1), the loading capacity (the weight ratio of PFD to MPDA) was calculated as 53% by measuring the PFD@MPDA absorbance at 319 nm. It is reported that the loading efficiency of MPDA is better than PDA without mesoporous (Chen et al., 2016; Peng et al., 2019), the satisfying loading efficiency of PFD@MPDA in our study may also be thanks to the large specific surface area brought by the mesoporous structure.

*In vitro*, the release of PFD from PFD@MPDA was investigated in PBS by a UV-Vis spectrophotometer. PFD released from PFD@MPDA was accumulated in a neutral solution (pH 7.4) mimicking *in vivo* environments. After 72 h, about 22% of PFD is released from PFD@MPDA at pH 7.4 (Figure 1H).

### 3.3 Cytotoxicity and Blood Compatibility

**FIGURE 2:**
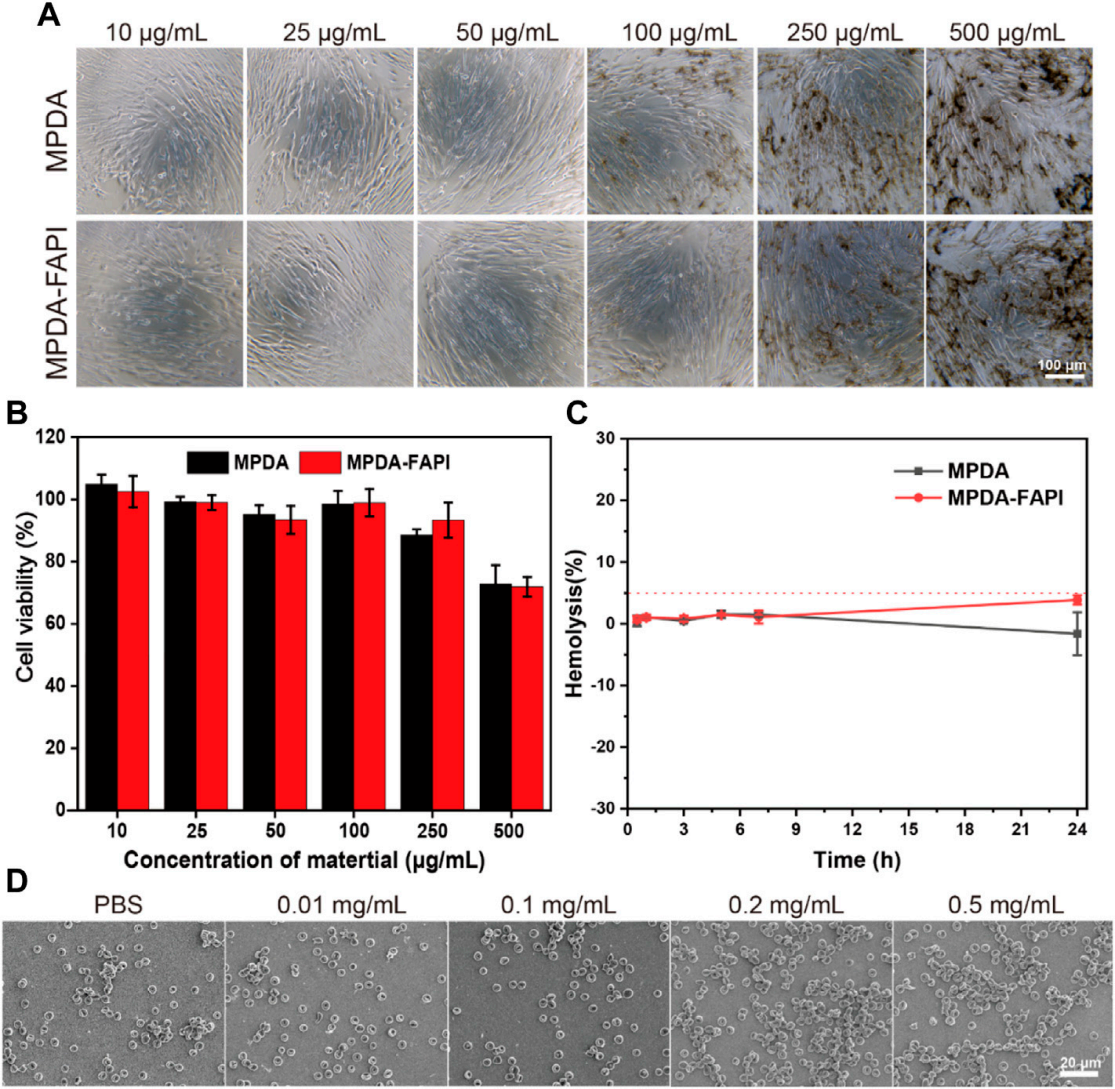
**(A,B)** Cytotoxicity test of MPDA and MPDA-FAPI NPSs after incubation with HFL1 cells for 24 h. **(C)** Hemolysis percentage of red blood cells at 0.01 mg/ml of MPDA and MPDA-FAPI. **(D)** Morphology of red blood cell incubated with MPDA-FAPI.

Biocompatibility is crucial for nanoparticles. Both MPDA and MPDA-FAPI nanoparticles of various concentrations (10–500 μg/ml) display negligible cytotoxicity after 24 h incubation with HFL1 cells. (Figures 2A,B), which proved that the nanoparticle does not harm normal HFL1 cells. After 72 h treated with MPDA and MPDA-FAPI (0.01 mg/ml), the hemolysis rate was less than 5% *in vitro* (Figure 2C). Also, the SEM result of RBC treated with MPDA-FAPI at various concentrations (10–500 μg/ml) shows normal morphology, consistent with the low hemolysis (Figure 2D). As expected, the biocompatibility of nanoparticle based on PDA is good because PDA is also the primary pigment of melanin, which naturally exist in the human body. Good biocompatibility allows nanoparticle utility in practice.

### 3.4 Cellular Uptake and Localization of Nanoparticle

**FIGURE 3:**
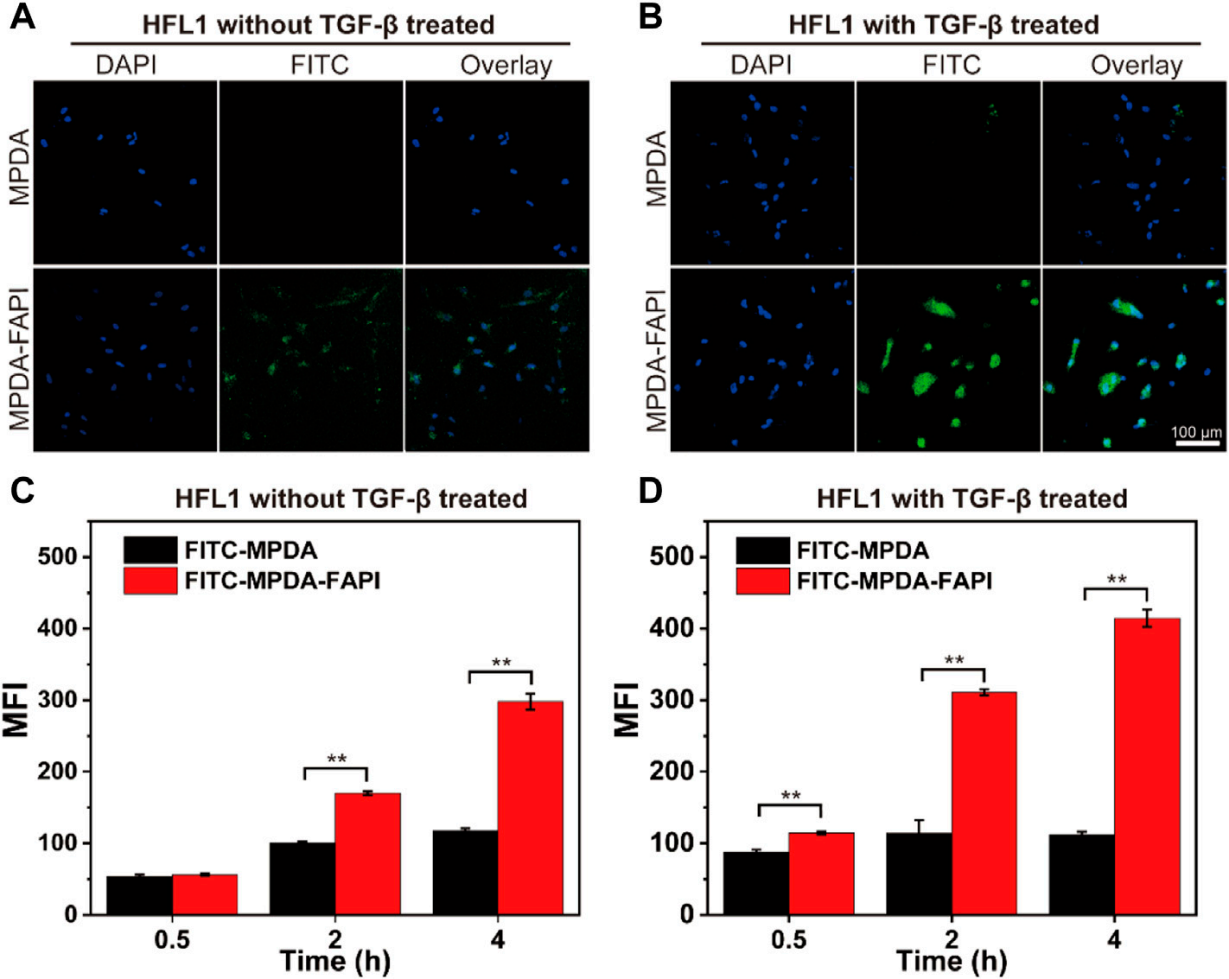
**(A,B)** CLSM images of HFL1 cells (treated without/with TGF-β) uptake MPDA and MPDA-FAPI for 4 h. **(C,D)** Flow cytometer result of HFL1 cells (treated without/with TGF-β) uptake of MPDA and MPDA-FAPI (**p < 0.01).

We observed the HFL1 treated without/with TGF-β uptake MPDA and MPDA-FAPI for 4 h by the confocal laser scanning microscope (CLSM), the highest cell uptake was observed for FITC-MPDA-FAPI in HFL1 with TGF-β treated (Figures 3A,B). The cellular uptake of MPDA and MPDA-FAPI for 0.5, 2, and 4 h were quantificationally investigated by a flow cytometer (Figures 3C,D). With the time changed, the uptake of nanodrug increased, and at each time point, the MFI of the FITC-MPDA-FAPI group is higher than FITC-MPDA no matter whether HFL1 was treated by TGF-β or not. We also found that the uptake of FITC-MPDA-FAPI is higher in HFL1 which is treated by TGF-β than the nontreated group, but the difference between FITC-MPDA uptake in HFL1 treated by TGF-β and nontreated groups was tiny (Supplementary Figure S2). As expected, the HFL1 treated with TGF-β and incubated with FITC-MPDA-FAPI for 4 h showed the highest MFI (414 ± 12), which is consistent with the CLSM result.

The higher uptake of MPDA-FAPI by HFL1 treated with TGF-β indicates that the MPDA-FAPI is successfully targeting the activated fibroblast and improving the uptake of nanodrug, which is beneficial for pulmonary fibrosis foci target drug delivery.

### 3.5 *In Vitro* Therapy of Nanoparticle

TGF-β is considered a key regulator in tissue fibrosis by promoting the overexpression of downstream fibrosis-related target genes. The HFL1 is activated *in vitro* and treated by TGF-β for 48 h, which can promote the proliferation and migration of HFL1, and also promote the expression of the biomarkers of fibrosis (such as FN and α-SMA). PFD can inhibit the activity of TGF-β and regulate the downstream signal of the TGF/Smad signal pathway, which has the properties of antifibrosis. Compared to pure PFD and PFD@MPDA, PFD@MPDA-FAPI can promote the drug uptake of HFL1, which manifest the best antifibrosis properties.

**FIGURE 4:**
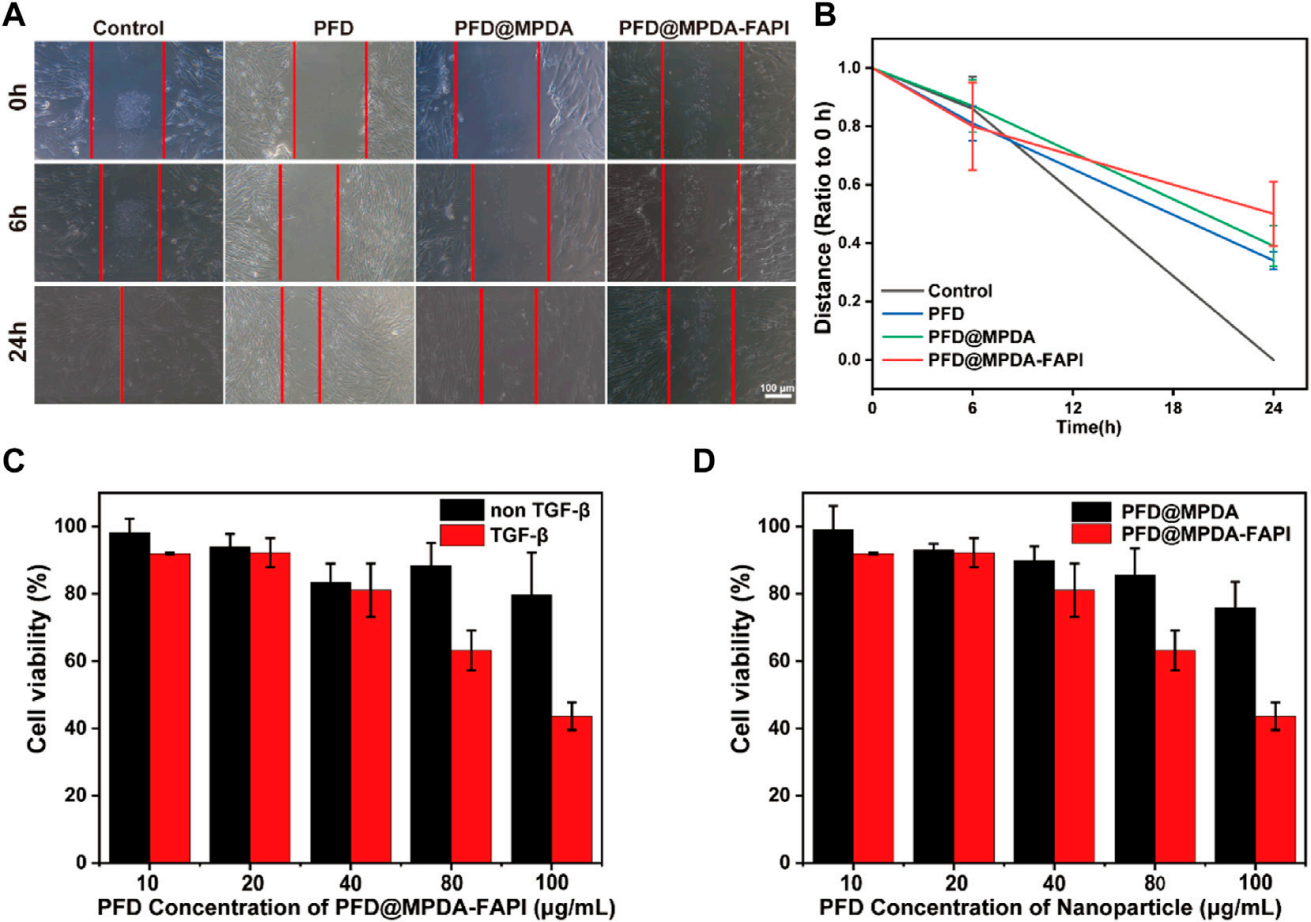
**(A,B)** Scratch test results of HFL1-1 treated by TGF-β and incubated with PFD, PFD@MPDA, and PFD@MPDA-FAPI. **(C)** Proliferation test of PFD@MPDA-FAPI NPSs after incubation with HFL1 cells without/with TGF-β for 24 h. **(D)** Proliferation test of PFD@MPDA and PFD@MPDA-FAPI NPSs after incubation with HFL1-1 cells treated by TGF-β for 24 h (**p < 0.01).

The effects of NPS-loaded PFD on the proliferation of HFL1 were analyzed by MTT assay. The results showed that PFD@MPDA-FAPI could inhibit the proliferation of HFL1 treated by TGF-β, while HFL1 without TGF-β treatment had not been significantly inhibited (Figure 4C). In addition, PFD@MPDA-FAPI performs better than PFD@MPDA in inhibiting the proliferation of HFL1 treated with TGF-β (Figure 4D). According to the results of the MTT assay, the 100 μg/ml PFD concentration of MPDA@PFD and MPDA@PFD-FAPI were chosen for the scratch test to measure HFL1 migration. The results showed that both PFD, PFD@MPDA, and PFD@MPDA-FAPI could inhibit the migration of HFL1 treated by TGF-β, and PFD@MPDA-FAPI performed best (Figures 4A,B).

The study suggested that PFD@MPDA-FAPI targeting FAP does better than PFD@MPDA on inhibitor the proliferation and migration of activated fibroblast, and had a tiny effect on the normal fibroblast, suggesting that nanoparticle target to FAP is useful in the therapy of pulmonary fibrosis.

### 3.6 *In Vivo* Biodistribution

**FIGURE 5:**
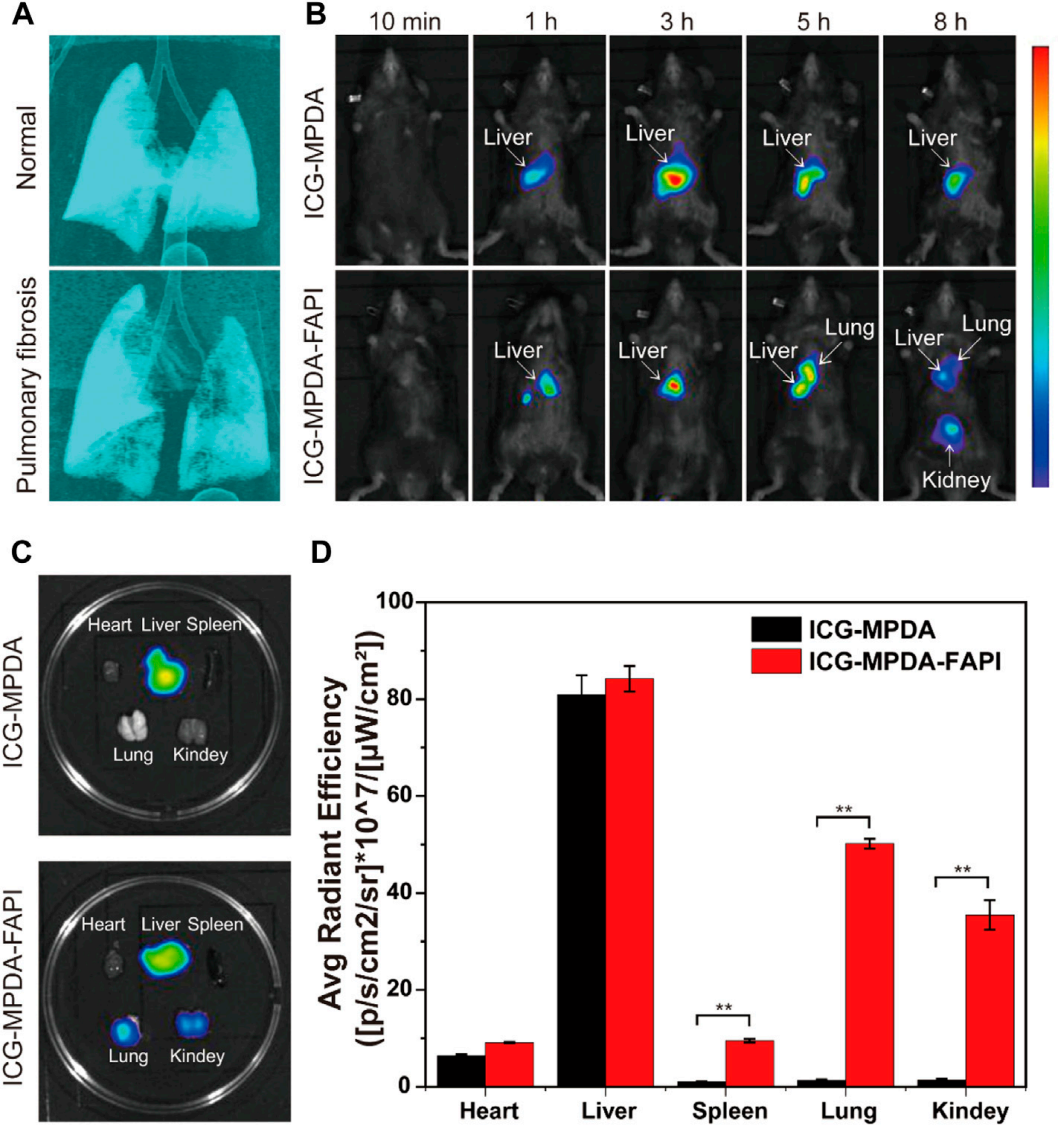
**(A)** 3D CT of the normal lung and pulmonary fibrosis. **(B)** ICG-MPDA and ICG-MPDA-FAPI NIR fluorescence imaging *in vivo* of pulmonary fibrosis mice. **(C)** ICG-MPDA and ICG-MPDA-FAPI NIR fluorescence imaging of pulmonary fibrosis mice *in vitro*. **(D)** ICG-MPDA and ICG-MPDA-FAPI NIR fluorescence quantification of pulmonary fibrosis mice *in vitro*.

Pulmonary fibrosis mice were induced by BLM (2 mg/kg) administered transtracheal *via* a laryngoscope, and after 6 days, micro-CT was performed to confirm the pulmonary fibrosis, then the next day *in vivo* NIR imaging was performed to explore the biodistribution of MPDA and MPDA-FAPI in pulmonary fibrosis mice. The 3D remolding of lungs based on micro-CT showed that, compared to healthy pulmonary, the normal airway structure in fibrosis lungs is reduced (Figure 5A). Only MPDA-FAPI has a significant accumulation in the lungs of pulmonary fibrosis mice (Figure 5B). After 8 h imaging, the major organs of mice were resected and quantified the radiant efficiency, the lung of the pulmonary fibrosis mouse injected with ICG-MPDA-FAPI also showed a high radiant efficiency, which was consistent with the *in vivo* ICG imaging (Figures 5C,D).

The ICG-MPDA and ICG-MPDA-FAPI were first observed in livers because the liver is a typical organ with the endothelial reticular system, in which Kupffer cells uptake nanoparticles. For ICG-MPDA, the accumulation only lasted in the liver; as for ICG-MPDA-FAPI, the nanoparticle gradually accumulates in fibrosis lung, in which FAP is overexpressed. At a later point, ICG-MPDA-FAPI accumulates in the kidneys because the hydrophilic PEG brought by the NH_2_-PEG_2_-FAPI may change the pharmacokinetic. The NIR imaging indicated the MPDA-FAPI is targeted to the overexpress FAP of fibrosis foci in *in vivo*, MPDA-FAPI is a successful FAP targeted nanodelivery carried out in *in vivo*.

### 3.7 *In Vivo* Antifibrosis Properties

**FIGURE 6:**
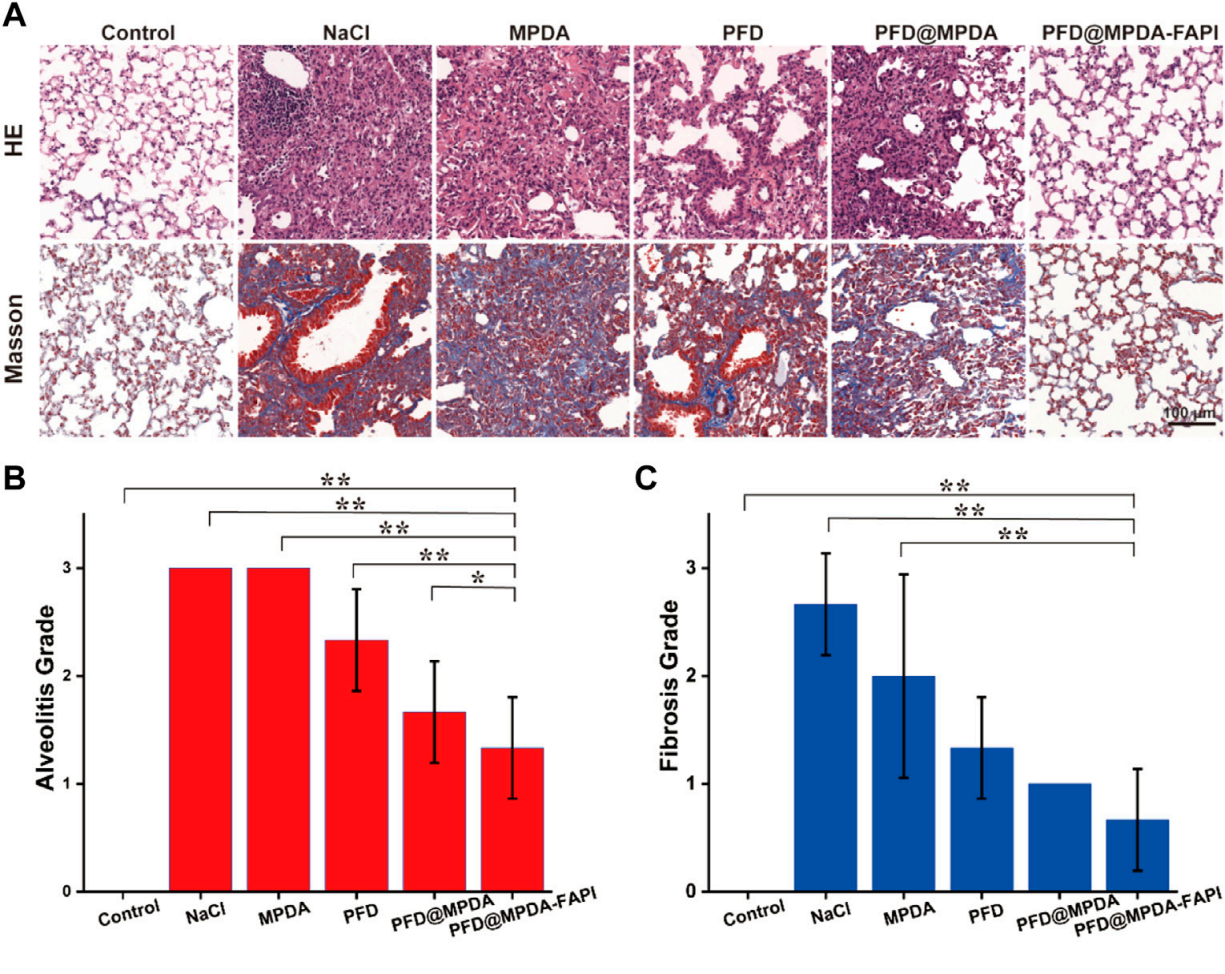
**(A)** HE staining and Masson staining of lungs among different treatment groups. **(B,C)** Grade of alveolitis and fibrosis among different treatment groups.

After the nanodrug treatment, the weight of mice varies mild, which indicates the nanodrug is safe (Supplementary Figure S6). The grades of alveolitis and fibrosis of the mice in the control group were both 0. Compared to the control group, NaCl, MPDA, and PFD@MPDA groups, the PFD@MPDA-FAPI shows the lowest grades of alveolitis and fibrosis (Figures 6A–C).

**FIGURE 7:**
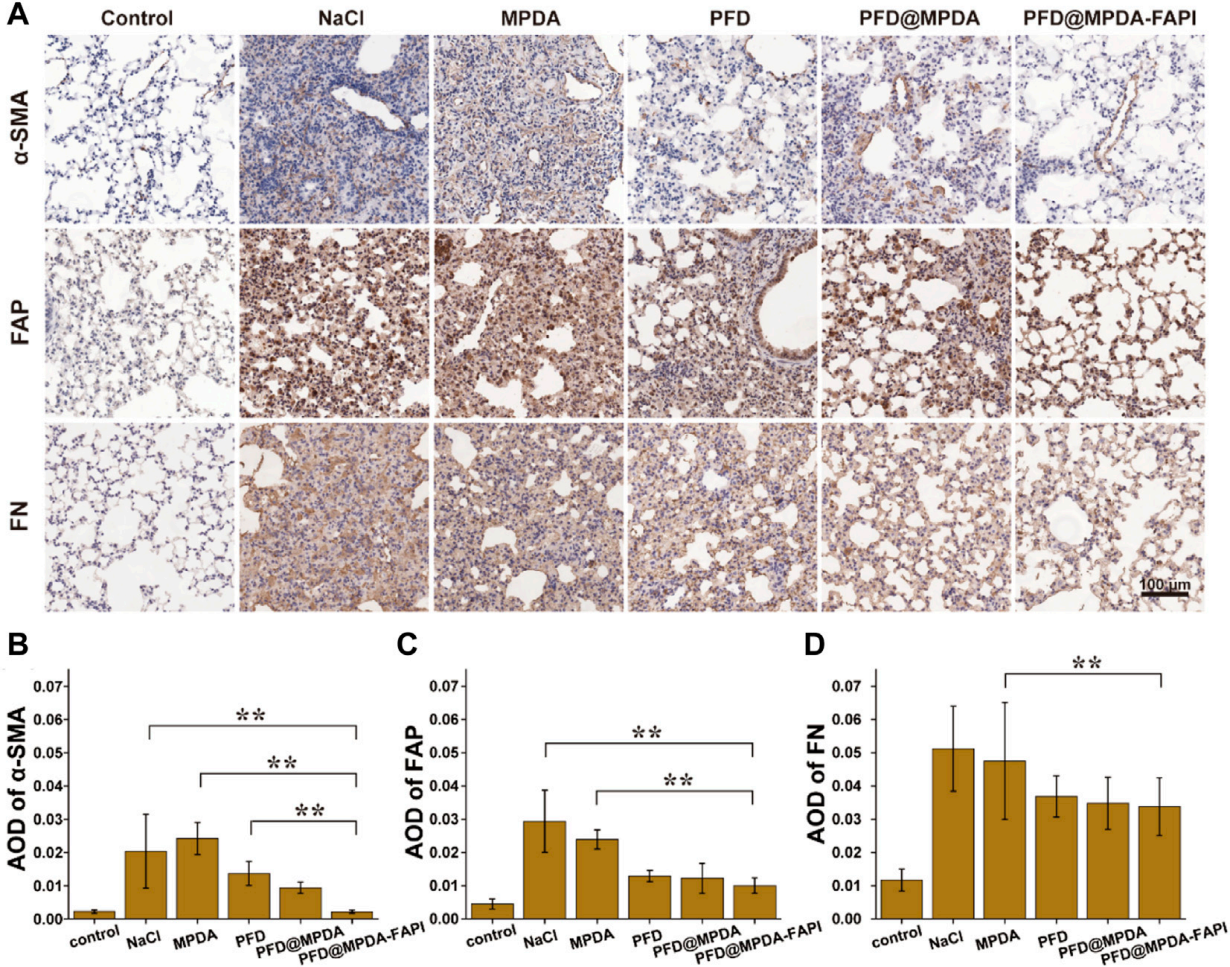
**(A)** IHC staining for FN, FAP, and α-SMA among different treatment groups. **(B–D)** AOD of IHC staining for FN, FAP, and α-SMA among different treatment groups.

In IHC staining, the control group showed the lowest expression of α-SMA, FAP, and FN. Compared to the control group, the expression of α-SMA, FAP, and FN were increased in NaCl and MPDA groups. The expression of α-SMA, FAP, and FN in PFD, PFD@MPDA, and PFD@MPDA-FAPI groups decreased compared to NaCl and MPDA groups, and the PFD@MPDA-FAPI group manifested the lowest expression (Figures 7A–D).

**FIGURE 8:**
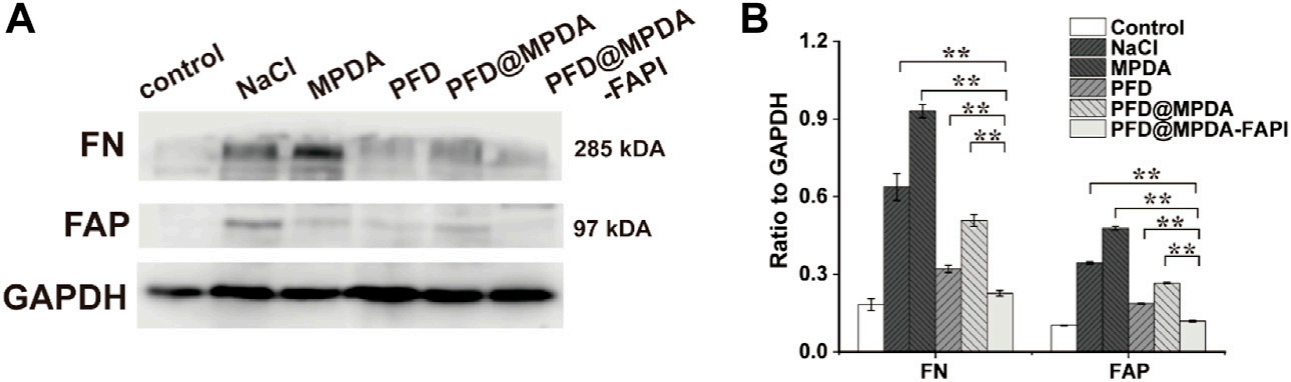
**(A,B)** WB results of FN and FAP among different treatment groups (*p < 0.05).

The results of WB were generally consistent with the IHC staining. In WB analyses, the control group showed a low expression of FAP and FN. Compared to the control group, NaCl and MPDA groups show higher expression of FN and FAP. In the PFD@MPDA-FAPI group, the expression of FN and FAP were lower than in the PFD and PFD@MPDA groups, which indicated that the antifibrosis properties of PFD@MPDA-FAPI are the best (Figures 8A,B).

In the pathological produce of pulmonary fibrosis, alveolitis comes first, then the fibroblast is activated, expressed FAP, and then activated fibroblast differentiate to myofibroblast, which is a key cell-expressed α-SMA to produce excessive exocellular matrix (Yu and Tang, 2022). The excessive exocellular matrix is comprised of collagen fibers, which can be determined by Masson staining and FN IHC.

Therefore, the WB and IHC results of low FN, FAP, and α-SMA expression in the PFD@MPDA-FAPI group, indicate that PFD@MPDA-FAPI shows good antifibrosis properties *in vivo* and performed better than pure PFD and PFD@MPDA in generally.

We also analyzed the antifibrosis properties of nanodrug after six times treatments. The PFD@MPDA-FAPI also performed the best, which is similar to the result of three times of treatments (Supplementary Figures S3, S4). It is considered that the pulmonary fibrosis of mice induced by BLM may change with time, in general, the fibrosis lesion may subside and is close to normal at the end, (Degryse and Lawson, 2011) so it is hard to explain the difference between three times and six times treatment groups, but either three times or six times treatment, we can observe the pleasing antifibrosis properties of PFD@MPDA-FAPI.

## 4 Conclusion

PFD@MPDA-FAPI, a nanoparticle developed based on MPDA, loading PFD, and targeting FAP on activated fibroblast, performs better than pure PFD and MPDA@PFD in inhibiting the proliferation and migration of the activated fibroblast, which is crucial in antifibrosis therapy, and do no harm to normal fibroblast. The nanoparticle is safe for IV administration and can accumulate in the fibrosis lung *in vivo*, also manifesting a good antifibrosis property. PFD@MPDA-FAPI is a promising nanodrug for antifibrosis therapy without the side effects of the stomach caused by PFD orally administrated. In addition, given consideration that FAP is also overexpressed on cancer-associated fibroblast, the MPDA also manifests a photothermal property, so replacing the loading drug with an anticancer drug may also develop a new tumor target nanodrug, which may perform well in cancer therapy combined with chemotherapy and photothermal therapy.

## Data Availability

The original contributions presented in the study are included in the article/Supplementary Material; further inquiries can be directed to the corresponding authors.
