# Supplementary material for: Mesoporous Polydopamine Loaded Pirfenidone Target to Fibroblast Activation Protein for Pulmonary Fibrosis Therapy
Source: Front Bioeng Biotechnol. 2022 Jul 22;10:920766. doi: 10.3389/fbioe.2022.920766 (PMC9363109; doi:10.3389/fbioe.2022.920766)
Supplement: Supplementary file 1 [file DataSheet1.pdf]

Supplementary information

Mesoporous Polydopamine loaded Pirfenidone Target to Fibroblast Activation Protein for Pulmonary Fibrosis Therapy

Qi Fang[1]，Shaoyu Liu[1], Ruiyue Zhao[1], Peng Hou[1], Youcai Li[1], Jie Lv[1], Xiaoyao Zhang[1], and Xinlu Wang[1*]

[1]Department of Nuclear Medicine, First Affiliated Hospital of Guangzhou Medical University

[*]Correspondence: Xinlu Wang (71Lu@163.com)

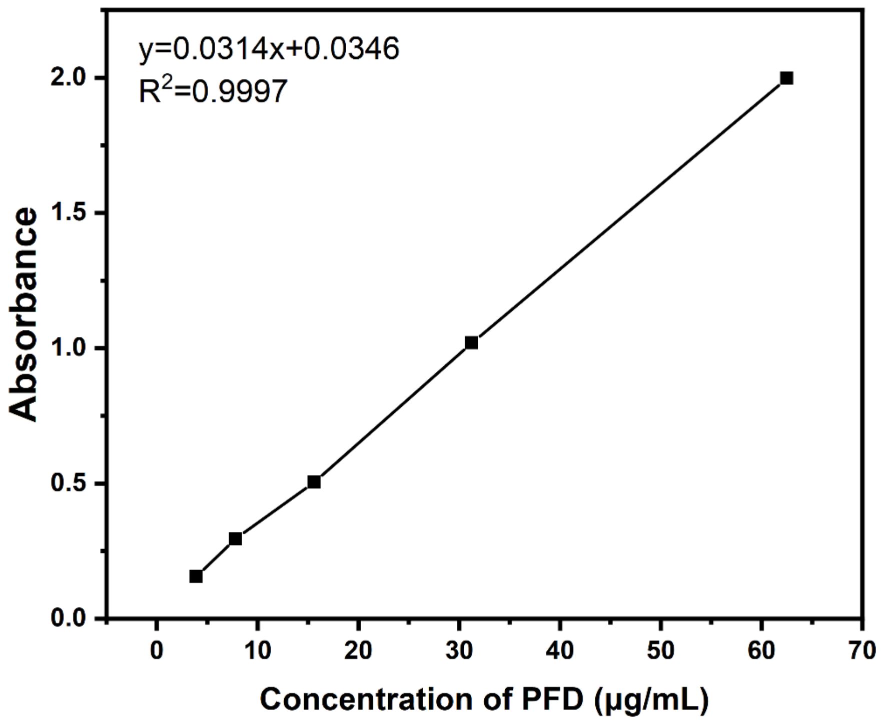

Figure S1. Standard curve of PFD.

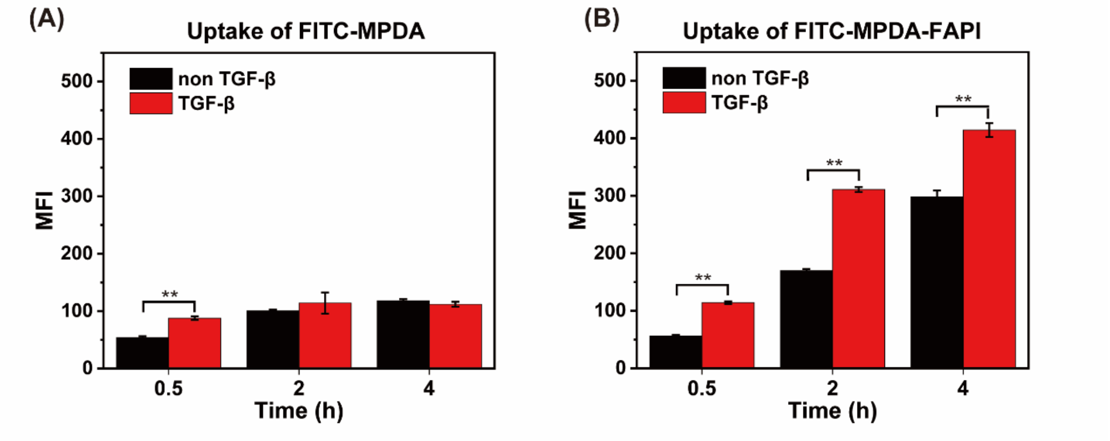

Figure S2. (A) FITC-MPDA and (B) FITC-MPDA-FAPI uptake in in HFL1.(**p＜0.01)

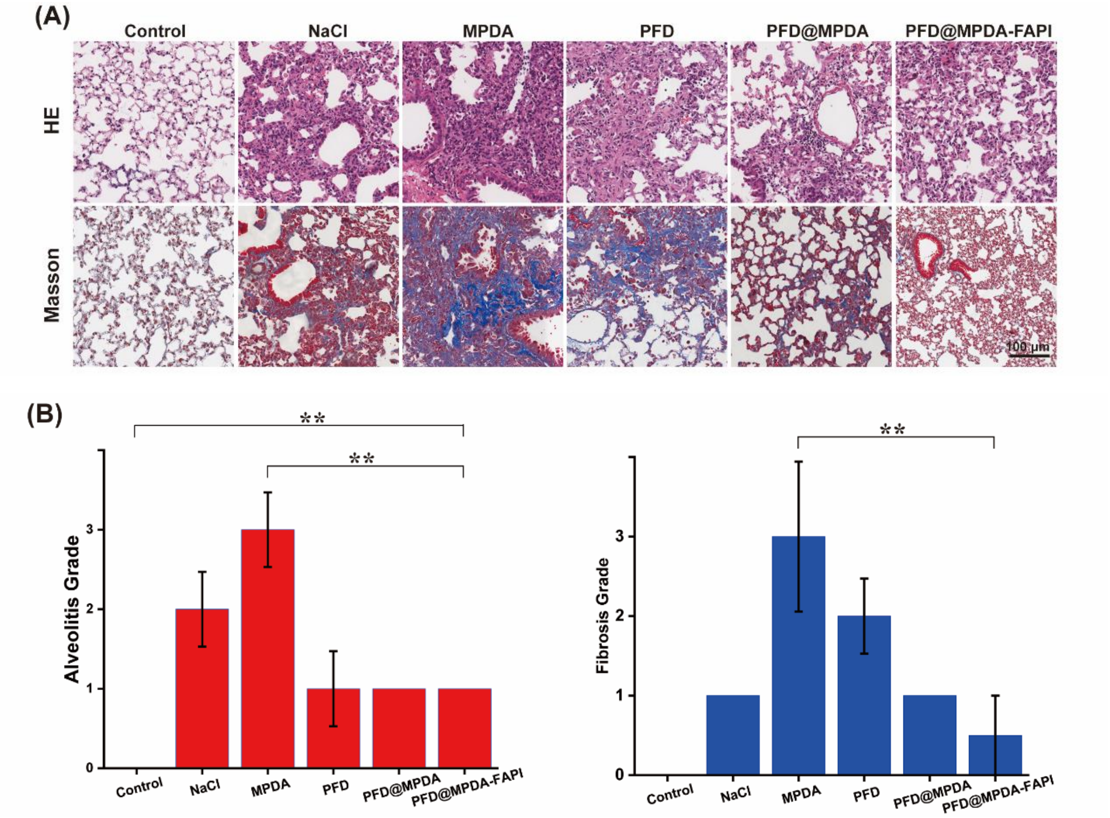

Figure S3. (A) HE staining and Masson staining of lungs among different treatment groups. (B) Grade of alveolitis and fibrosis among different treatment groups.

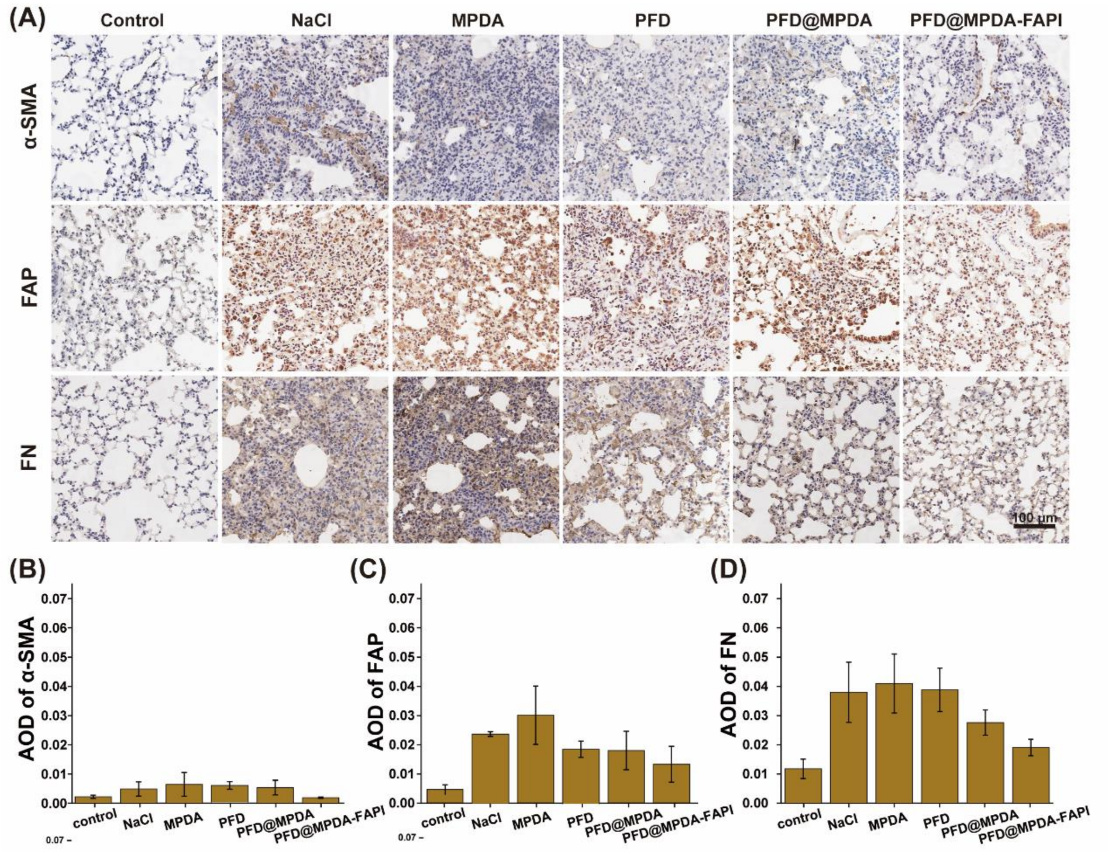

Figure S4. (A) IHC staining for FN, FAP, and α-SMA among different treatment groups. (B), (C), and (D) AOD of IHC staining for FN, FAP, and α-SMA among different treatment groups.

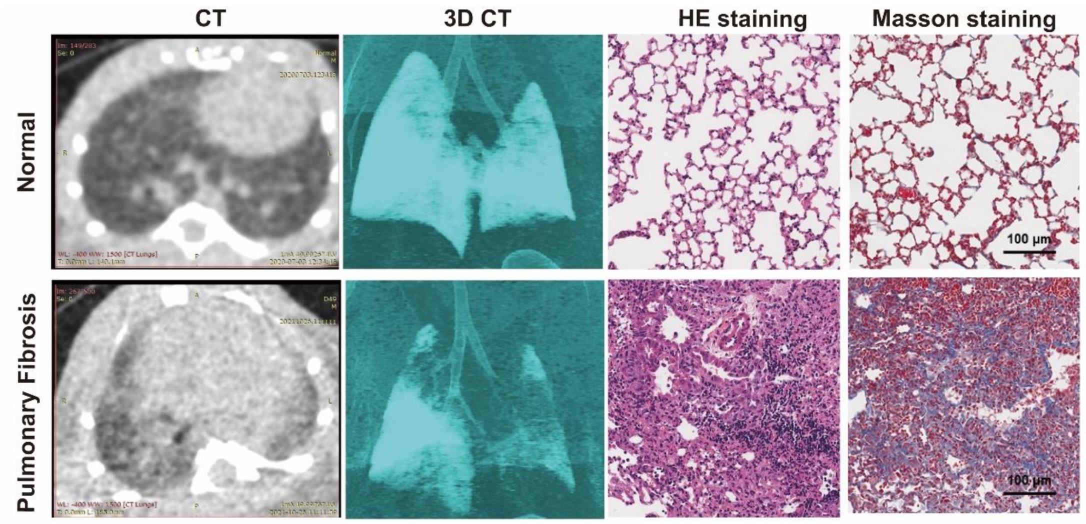

Figure S5. CT，3D CT，HE staining and Masson staining images of normal mouse and pulmonary fibrosis mouse induced by BLM .

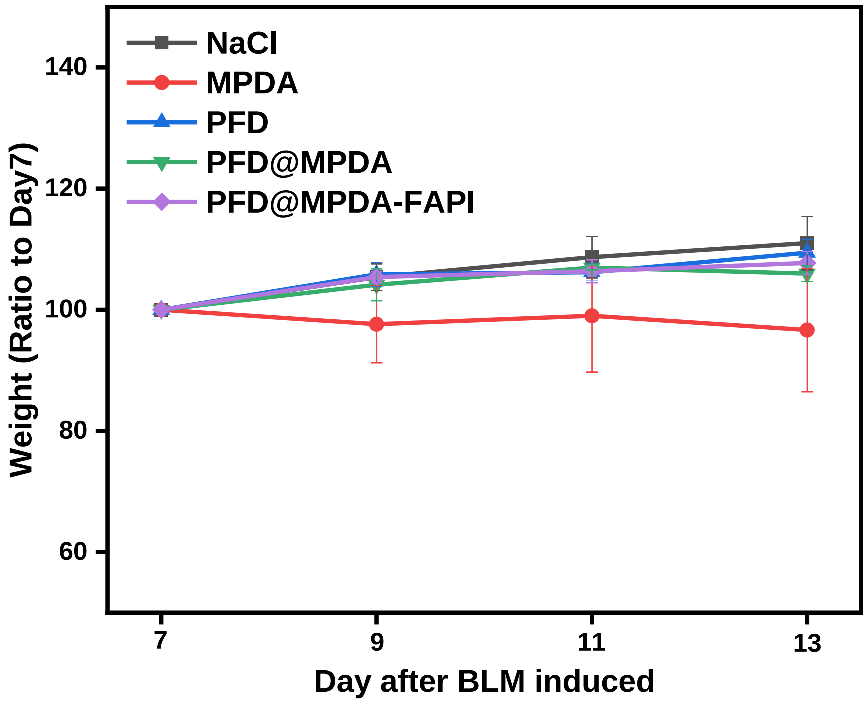

Figure S6. Weight of pulmonary fibrosis mice during 3 times treatment.
